# Pineal Gland Metastasis From Poorly Differentiated Carcinoma of Unknown Primary Origin

**DOI:** 10.3389/fendo.2020.597773

**Published:** 2020-10-23

**Authors:** Joshua A. Cuoco, Michael W. Kortz, Michael J. Benko, Robert W. Jarrett, Cara M. Rogers, Mark R. Witcher, Eric A. Marvin

**Affiliations:** ^1^ Section of Neurosurgery, Carilion Clinic, Roanoke, VA, United States; ^2^ Virginia Tech Carilion School of Medicine, Roanoke, VA, United States; ^3^ School of Neuroscience, Virginia Polytechnic Institute and State University, Blacksburg, VA, United States; ^4^ Department of Neurosurgery, University of Colorado, Aurora, CO, United States; ^5^ College of Osteopathic Medicine, Kansas City University, Kansas City, MO, United States; ^6^ Department of Pathology, Carilion Clinic, Roanoke, VA, United States

**Keywords:** pineal gland, neuroendocrinology, cancer endocrinology, carcinoma of unknown primary (CUP), metastasis

## Abstract

Pineal metastasis is an exceedingly rare finding in patients with systemic malignancies. Such lesions are typically the manifestation of a primary lung cancer; nonetheless, a variety of malignancies have been reported to disseminate to the pineal gland including gastrointestinal, endocrine, and skin cancers, among others. However, to our knowledge, pineal gland metastasis without a primary origin has yet to be described. Carcinoma of unknown primary origin is a heterogeneous group of cancers characterized by the presence of metastatic disease without an identifiable primary tumor on metastatic workup. Here, we present a case of a 65-year-old male found to have a heterogeneously enhancing lesion of the pineal gland as well as an enhancing lesion of the left cerebellar hemisphere. Comprehensive metastatic workup demonstrated multifocal metastatic adenopathy without an identifiable primary lesion. Stereotactic biopsy of the pineal lesion revealed poorly differentiated carcinoma with an immunophenotype most consistent with gastrointestinal origin. To our knowledge, this is the first case to describe a pineal gland metastasis without a primary origin. We discuss the relevant literature on pineal gland metastases as well as carcinoma of unknown primary origin.

## Background

Pineal region tumors are a rare entity constituting approximately 1% of all intracranial tumors in the adult population (1). Although metastasis to the brain is common in the setting of primary malignancies, metastasis specifically to the pineal region is an exceedingly rare phenomenon accounting for 0.4–3.8% of all intracranial metastases (1, 2). Metastasis to the pineal gland is most commonly a derivative of a primary lung malignancy; nevertheless, there are reports of a variety of primary tumors that have metastasized to this neuroendocrine secretory circumventricular organ including esophageal, stomach, liver, colon, pancreas, kidney, bladder, prostate, thyroid, breast, melanoma, myeloma, and leukemia (3, 4). However, to the best of the authors’ knowledge, pineal gland metastasis without a primary origin has yet to be described.

Carcinoma of unknown primary origin (CUP) is a heterogeneous group of cancers defined by the presence of metastatic disease without an identifiable primary tumor on metastatic workup (5, 6). CUP has been reported to constitute 2–5% of all cancer cases and, remarkably, represents up to 15% of all patients with brain metastases (5, 6). Here, we present a case of a 65-year-old male found to have a heterogeneously enhancing lesion of the pineal gland as well as an enhancing lesion of the left cerebellar hemisphere. Metastatic workup including computed tomography (CT) of the chest, abdomen, and pelvis as well as whole-body positron emission tomography (PET) scan demonstrated multifocal metastatic adenopathy without an identifiable primary lesion. Stereotactic biopsy of the pineal lesion revealed poorly differentiated carcinoma with an immunophenotype most consistent with gastrointestinal origin. To our knowledge, this is the first case to describe a pineal gland metastasis from CUP. We discuss the relevant literature on pineal gland metastases as well as carcinoma of unknown primary origin.

## Case Presentation

**Figure 1:**
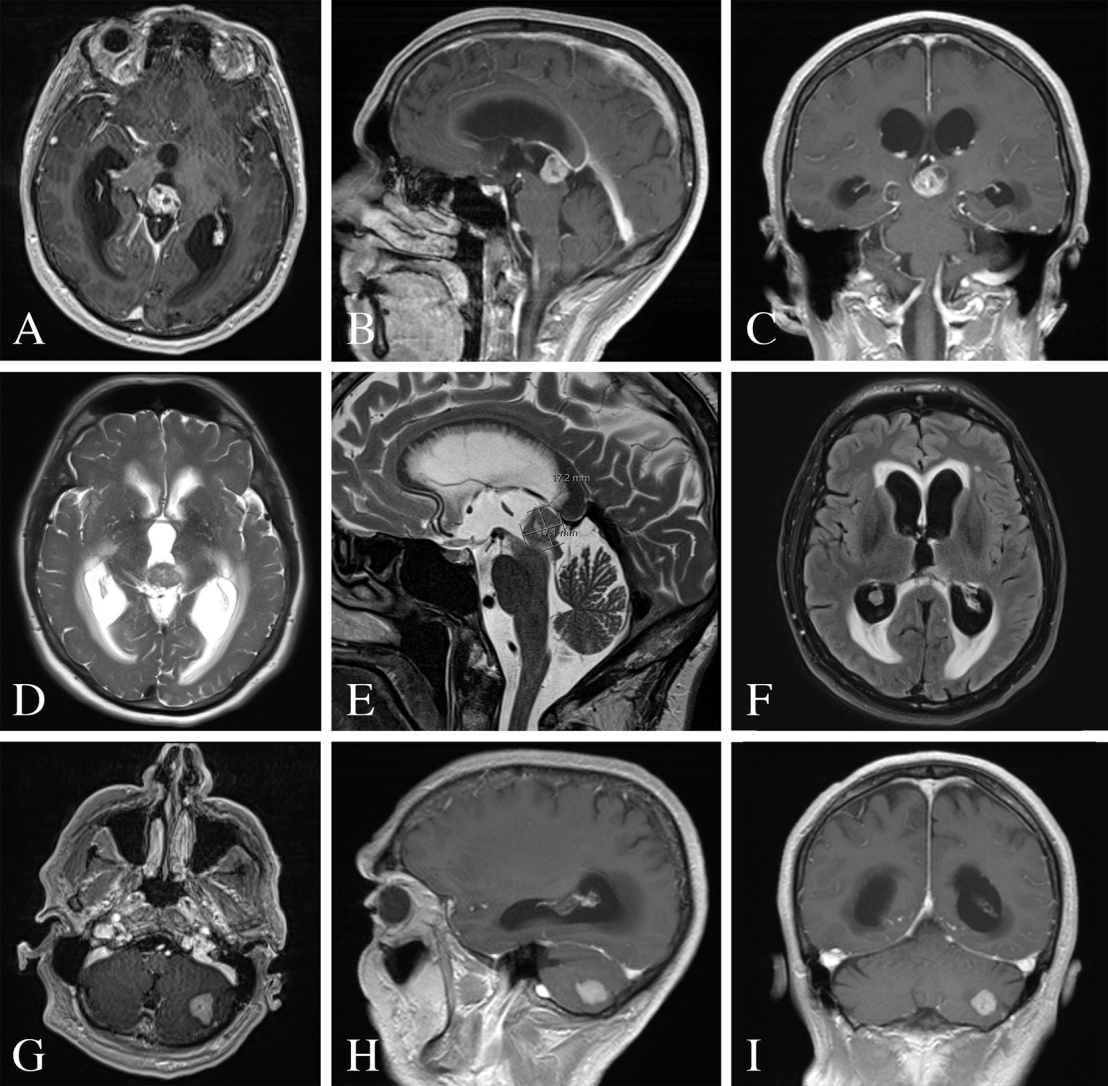
Pre-operative MRI of the brain. **(A–C)** MRI T1-weighted images with contrast demonstrating a heterogeneously enhancing lesion of the pineal gland. **(D, E)** MRI T2-weighted images with a T2 hypointense lesion with mass effect and compression of the cerebral aqueduct resulting in supratentorial ventricular dilatation and periventricular white matter signal abnormality. **(F)** MRI FLAIR image demonstrating periventricular transependymal flow of cerebrospinal fluid indicative of acute hydrocephalus. **(G–I)** MRI T1-weighted images with contrast demonstrating an enhancing lesion of the left cerebellar hemisphere.

A 65-year-old gentleman presented to our emergency department with two weeks of progressively worsening headaches and fatigue. Physical examination was unremarkable. The patient was without any relevant past medical history or cancer diagnoses. He admitted to a 60-pack year smoking history. CT of the head demonstrated a partially calcified hyperdense pineal lesion causing obstructive hydrocephalus with marked supratentorial ventricular dilatation. Subsequently, magnetic resonance imaging (MRI) revealed a heterogeneously enhancing lesion of the pineal gland with mass effect and compression of the cerebral aqueduct resulting in supratentorial ventricular dilatation with periventricular white matter T2 hyperintensity consistent with transependymal flow (**Figure 1**). Moreover, MRI revealed a second enhancing lesion of the left cerebellar hemisphere. Metastatic workup demonstrated multifocal metastatic adenopathy including the supraclavicular, prevascular mediastinal, paratracheal, hilar, and internal mammary regions. However, there was no evidence of a primary malignancy or other metastatic disease in the chest, abdomen, or pelvis. Tracheal aspirates retrieved *via* bronchoscopy demonstrated alveolar histiocytes, mixed inflammatory cells and reactive pneumocytes without evidence of malignant cells. The patient was started on dexamethasone and medically optimized for surgical intervention to address his symptomatic obstructive hydrocephalus and obtain tissue diagnosis.

**Figure 2:**
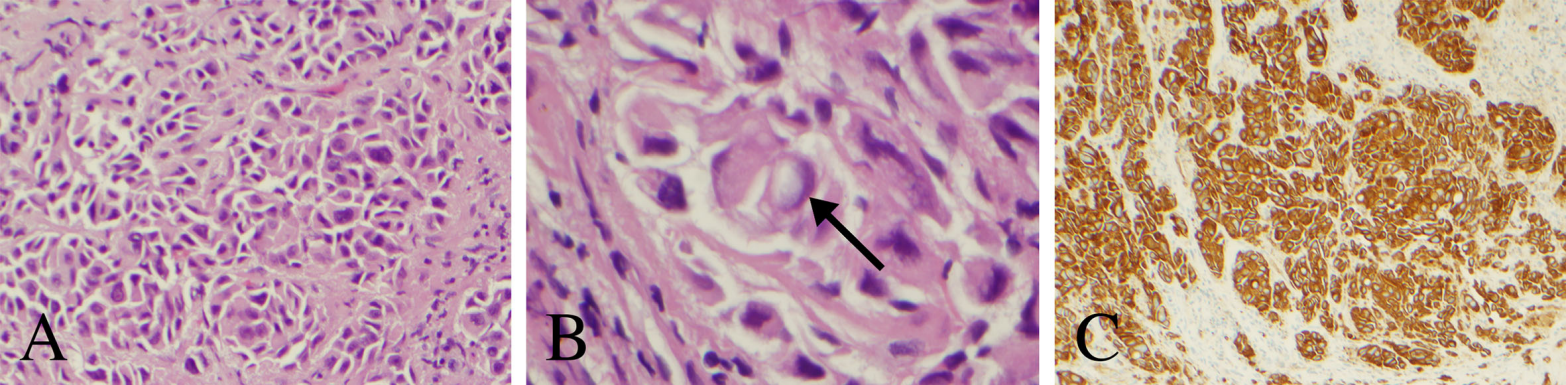
Histopathology and immunohistochemical analysis of the pineal lesion. **(A)** Hematoxylin and eosin stain with nests and singly dispersed cells with a high nuclear to cytoplasm ratio (200× magnification). **(B)** Hematoxylin and eosin stain demonstrating a signet ring-like cell (arrow) (600× magnification). **(C)** Cytokeratin AE1 immunostain demonstrating strong reactivity (100× magnification).

An endoscopic third ventriculostomy in conjunction with biopsy of the pineal lesion was performed with stereotactic navigation and intraoperative neuromonitoring (7). The patient tolerated the procedure well and without complication. Pathology was consistent with poorly differentiated carcinoma (**Figure 2**). The tumor consisted of nests and singly dispersed cells. A small minority of tumor cells exhibited signet ring features. The tumor cells were strongly and diffusely reactive for cytokeratin AE1/AE3, cytokeratin 7, and cytokeratin 20 immunohistochemical stains. p40 and PAX8 highlighted rare tumor cells. Cytokerain 5/6, napsin A, thyroid transcription factor-1, melan-A, prostate-specific antigen, CDX2, synaptophysin, glial fibrillary acidic protein and GATA3 immunostains were negative in all tumor samples. As such, the immunophenotype was most consistent with upper gastrointestinal or pancreatobiliary origin of a poorly differentiated carcinoma.

**Figure 3:**
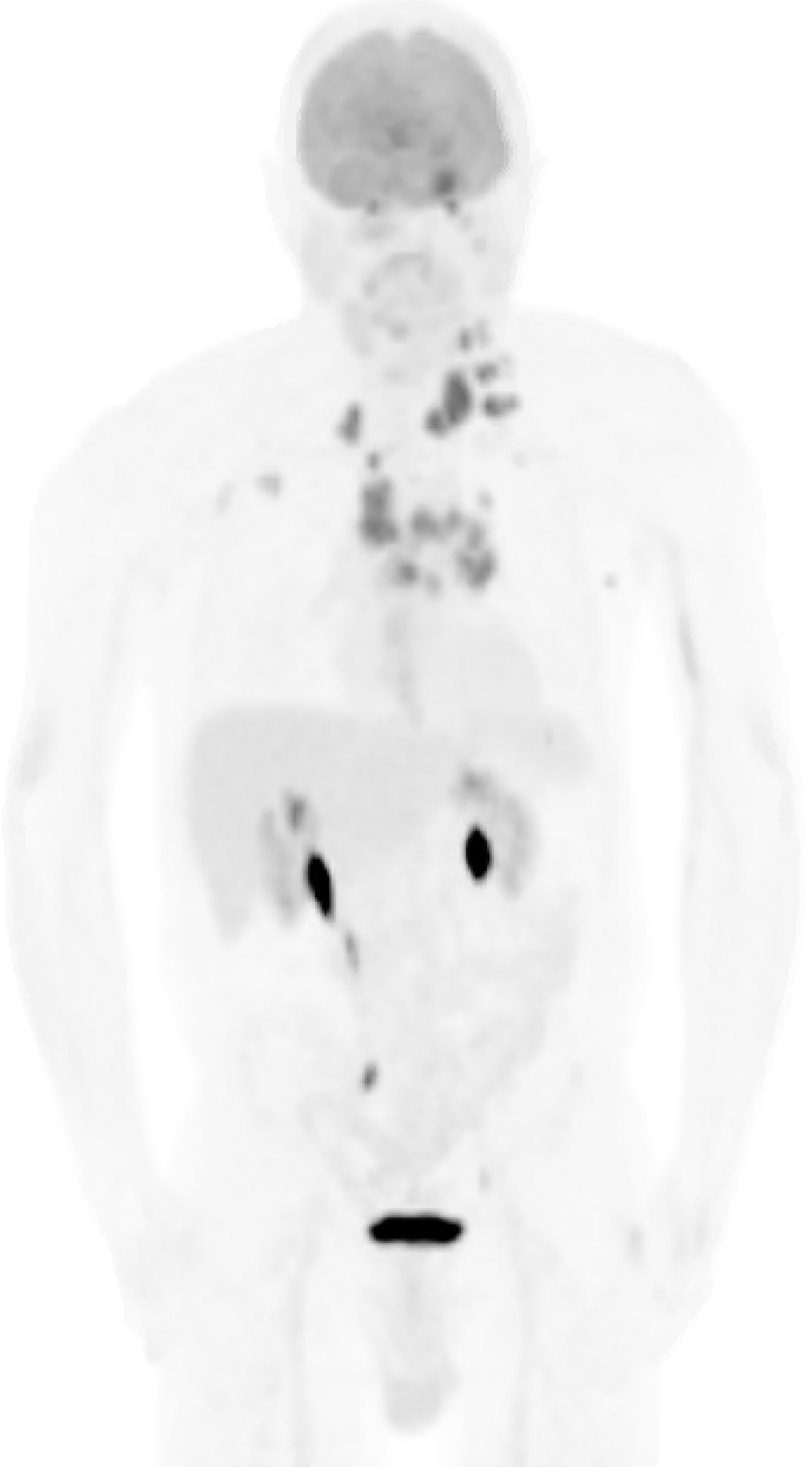
Whole-body PET scan. Numerous hypermetabolic lesions identified including the pineal region and left cerebellar hemisphere, bilateral cervical chain adenopathy, mediastinal/hilar adenopathy, bilateral axillary adenopathy, left cardiophrenic lymph node and left adrenal nodule. No evidence of a primary lesion was depicted.

**Figure 4:**
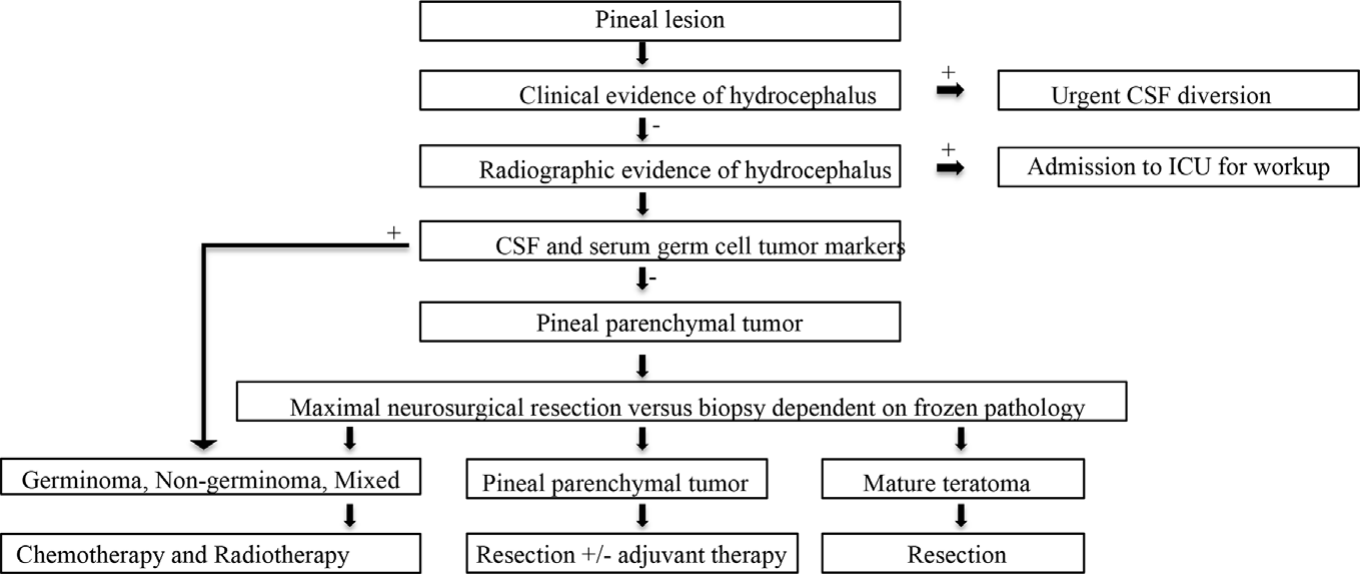
Basic diagnostic workup and treatment flowchart of a solitary pineal lesion.

**Figure 5:**
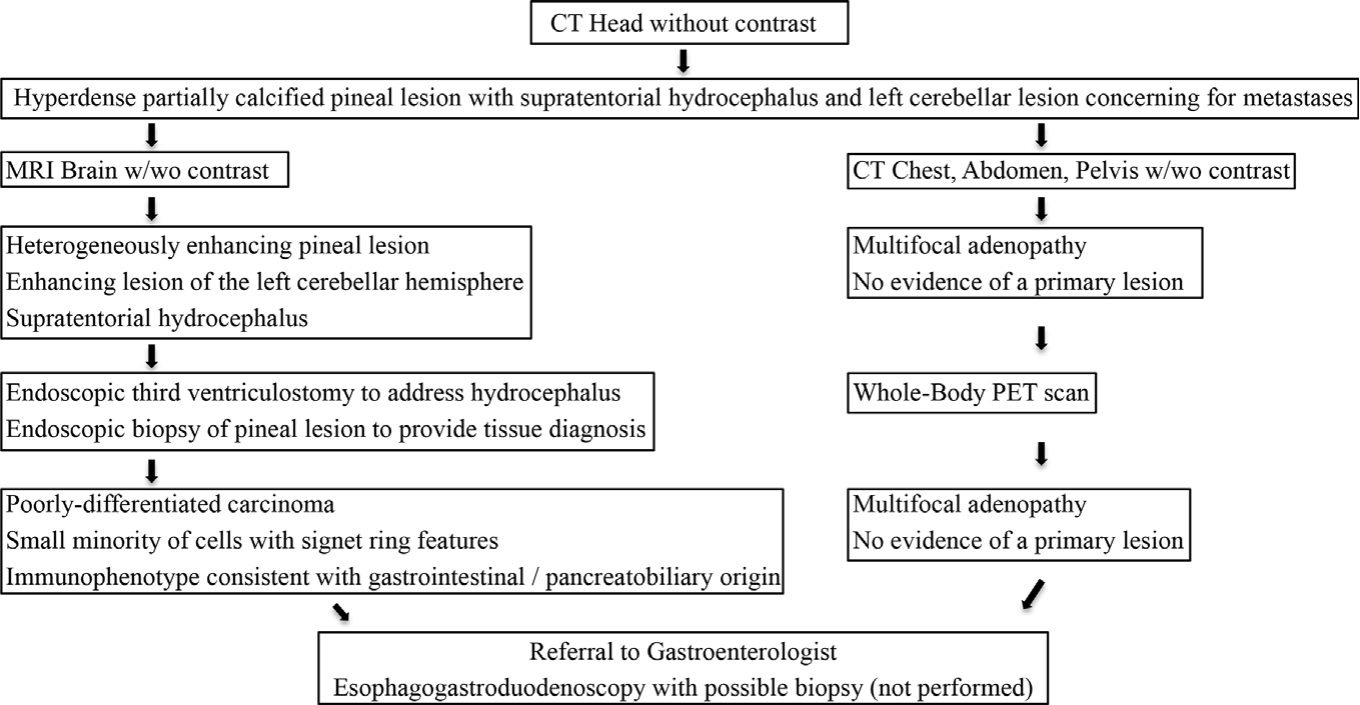
Our diagnostic workup and treatment plan of the case described herein (*i.e.*, suspected pineal region metastasis).

Post-operatively, the patient received CyberKnife radiosurgery directed at the pineal and left cerebellar metastases utilizing nine gray fractions over three treatment days for a total dose of 27 Gy. Prior to consideration of chemotherapeutic initiation, a PET scan was recommended for identification of the primary malignancy as well as staging of his disease. Imaging demonstrated hypermetabolism of the known multifocal metastatic adenopathy; however, no evidence of primary malignancy was revealed (**Figure 3**). Given the immunophenotype of the lesion and a small minority of tumor cells exhibiting signet ring features, the patient was referred to a gastroenterologist who recommended esophagogastroduodenoscopy with possible biopsy of any identifiable abnormal tissue. The patient declined further workup and treatment. He expired 3 months from the time of diagnosis due to respiratory issues. A basic diagnostic workup and treatment flowchart of a solitary pineal lesion without additional intracranial lesions on imaging is depicted in **Figure 4**. Our diagnostic workup and treatment plan of the case described herein (*i.e.*, suspected pineal region metastasis) is depicted in **Figure 5**.

## Discussion

The pineal gland, or epiphysis cerebri, represents an exceptionally uncommon site of systemic metastasis (1, 2). Indeed, metastasis to the pineal gland is most commonly a derivative of a primary lung malignancy; however, numerous other primary malignancies have been reported to metastasize to the epiphysis cerebri including esophageal, stomach, liver, colon, pancreas, kidney, bladder, prostate, thyroid, breast, melanoma, myeloma, and leukemia (3, 4). Considered one of the neuroendocrine secretory circumventricular organs, capillaries of the pineal gland are mostly permeable to solutes in the blood (8). As such, metastases are thought to spread hematogenously given the lack of the blood–brain barrier of the pineal region (9). Lesions of the pineal gland typically remain clinically silent until they meet a threshold in size to compress critical surrounding neuroanatomic structures causing diverse clinical symptomatology. Compression of the posterior aspect of the third ventricle and cerebral aqueduct may cause obstructive hydrocephalus and increased intracranial pressure manifesting clinically as progressive headaches, fatigue, and, ultimately, coma and death if left untreated. Moreover, compression of the dorsal midbrain, specifically the superior colliculus and rostral interstitial nucleus of medial longitudinal fasciculus, translates clinically into Parinaud’s syndrome characterized by upward gaze paralysis, pseudo-Argyll Robertson pupils, convergence-retraction nystagmus, eyelid retraction and conjugate downgaze in the primary position. Our patient presented with clinical sequelae attributable to hydrocephalus including progressive headaches and fatigue.

Cancer of unknown primary origin is defined as a group of metastatic tumors for which the standardized metastatic workup fails to discover the site of origin. The pathobiology of CUP remains to be elucidated; however, two hypotheses have been described to explain their inception. The first theory establishes that a tumor can develop without a premalignant lesion or primary tumor (10, 11). The second theory postulates that metastatic progression occurs parallel to development of the primary lesion emphasizing that CUP metastases may be a premature event in tumorigenesis (10, 11). Regardless of etiology, histological confirmation of a metastatic tumor is the fundamental basis of a diagnosis of CUP. Subsequent to light microscopy and immunohistochemistry, CUP can be classified in one of five morphological subtypes including: (i) well- or moderately differentiated adenocarcinoma, (ii) poorly differentiated adenocarcinoma or undifferentiated carcinoma, (iii) squamous-cell carcinoma, (iv) undifferentiated neoplasms, or (v) carcinomas with neuroendocrine differentiation (11). Subsequently, tumors with specific treatments must be excluded, such as lymphomas, germ-cell tumors, melanoma, or sarcoma. Further immunohistochemical analysis is then carried out on CUP cases. Our case was strongly and diffusely reactive with cytokeratin AE1/AE3 consistent with carcinoma and strongly positive for cytokeratin 7 and cytokeratin 20 typically consistent with upper gastrointestinal or pancreatobiliary origin. Further immunohistochemical stains including cytokerain 5/6, napsin A, thyroid transcription factor-1, melan-A, prostate-specific antigen, CDX2, synaptophysin, glial fibrillary acidic protein and GATA3 were negative. As such, the immunophenotype was most consistent with upper gastrointestinal or pancreatobiliary origin of a metastatic poorly differentiated carcinoma. The immunophenotype would not have been classic for melanoma, lung, prostate, kidney, bladder or lower gastrointestinal origin. Moreover, signet ring features are classically associated with signet ring cell carcinoma originating from the stomach, which further raised suspicion of a gastrointestinal origin of the pineal lesion (12).

CUP has traditionally been classified into two broad clinicopathologic groups with distinct outcomes. The first group includes patients with a favorable risk profile, more responsive to chemotherapy and long-term disease control (11, 13, 14). This group includes women with serous papillary adenocarcinoma of the peritoneal cavity, women with isolated axillary nodal adenocarcinoma, patients with midline poorly differentiated carcinoma, neuroendocrine carcinoma of unknown primary, squamous cell carcinoma involving cervical or inguinal lymph nodes, colonic type adenocarcinoma and men with prostate-specific antigen positive osteoblastic metastases (11, 13, 14). Comparatively, a second group exists who exhibit multiple visceral metastatic deposits, chemotherapy resistance and typically succumb to their disease within 6 months (11, 13, 14). This group, representing 80% of all CUP cases, encompasses metastatic adenocarcinoma to the liver, lungs, brain, or other viscera, non-papillary peritoneal adenocarcinoma and multiple prostate specific antigen null bony deposits (11, 13, 14). Despite the identification of these two clinicopathologic subgroups, the heterogeneity of CUP represents unpredictable objective responses to known chemotherapeutics (13).

Several studies have associated prognostic factors with poor patient survival in CUP including: male sex, Eastern Cooperative Oncology Group performance status >1, high comorbidities, age greater than 64 years of age, history of smoking (greater than 10 pack-years), weight loss, lymphopenia, low serum albumin, and elevated alkaline phosphatase and lactate dehydrogenase concentrations (11, 13, 14). Petrakis et al. examined factors from 311 patients with CUP and developed a novel prognostic scoring algorithm known as I-SCOOP (Ioannina Score for CUP Outpatient Oncologic Prognostication) based on clinicopathologic CUP subgroup, performance status, and presence or absence of leukocytosis (13). The clinicopathologic parameter encompassed three subgroups including (i) serous peritoneal, axillary nodal and squamous head and neck (zero points), (ii) nodal, neuroendocrine and mucinous peritoneal (one point), or (iii) visceral (two points) (11). The second parameter provided one point for leukocytosis (>10,000/mm^3^) and zero points for a normal white-blood cell count (<10,000/mm^3^) (13). The third parameter entailed performance status with one point for a performance status of one or greater (symptomatic and ambulatory, cares for self) and zero points for a performance status of zero (normal activity without restrictions) (13). Scores of zero, one, two, three, and four were associated with a median overall survival of 36, 14, 11, 8, and 5 months, respectively (13). The patient we present here was given a score of three based on this algorithm (two points for visceral, zero points for leukocytosis, one point for performance status) translating into a median overall survival of 8 months. Our patient expired 3 months after diagnosis.

Eighty percent of patients diagnosed with CUP exhibit a poor prognosis with a median overall survival of 6 months regardless of intervention (15). Empiric treatment of CUP includes combinatory chemotherapeutics, including platinum/taxane or platinum/gemcitabine, which has translated into response rates of approximately 20% with a median survival of 9 months (11, 13–15). A treatment regimen with carboplatin and paclitaxel has been used as first-line therapy with or without maintenance therapy with erlotinib and bevacizumab with a reported response rate of 53% and an overall survival of 13 months (15). In addition to chemotherapeutics, radiosurgery plays an integral role in the treatment of CUP. In our case, radiosurgery was offered for adjuvant treatment of the two intracranial lesions despite the lack of identification of a primary lesion. Han et al. retrospectively evaluated 540 patients who underwent gamma knife radiosurgery and demonstrated that identification of a primary tumor prior to the initiation of gamma knife radiosurgery did not affect patient outcomes (6). This emphasizes that radiosurgery can be an effective adjuvant treatment modality for brain metastases in cases without a primary lesion. Moreover, initiation of radiation should not be delayed pending identification of a primary source.

## Conclusions

Here, we describe the first case of a pineal gland metastasis from CUP. Albeit exceedingly rare, the presence of a pineal lesion in individuals with a known systemic malignancy should raise clinical suspicion for metastatic disease dissemination. However, as depicted by the present case, the pineal gland can also be a location for metastatic disease deposition with an unknown primary origin. With each reported case, we gain a better understanding of the natural history and therapeutic treatment options of carcinoma of unknown origin.

## Data Availability Statement

The original contributions presented in the study are included in the article/supplementary material. Further inquiries can be directed to the corresponding author.

## Ethics Statement

Written informed consent was obtained from the individual(s) for the publication of any potentially identifiable images or data included in this article.

## Author Contributions

JC, MK: primary authors of the manuscript. JC, MK, MB, RJ, CR, MW, EM: provided substantial contributions to the conception and design of the manuscript. JC, MK, MB, RJ, CR, MW, EM: contributed to manuscript revision, read and approved the submitted version. JC, MK, MB, RJ, CR, MW, EM: agree to be accountable for all aspects of the work ensuring that questions related to the accuracy or integrity of any part of the work are investigated and resolved. All authors contributed to the article and approved the submitted version.

## Conflict of Interest

The authors declare that the research was conducted in the absence of any commercial or financial relationships that could be construed as a potential conflict of interest.

## Abbreviations

CUP, carcinoma of unknown primary origin; CT, computed tomography; PET, positron emission tomography; MRI, magnetic resonance imaging.
